# Adaptation of CD8 T Cell Responses to Changing HIV-1 Sequences in a Cohort of HIV-1 Infected Individuals Not Selected for a Certain HLA Allele

**DOI:** 10.1371/journal.pone.0080045

**Published:** 2013-12-03

**Authors:** Julia Roider, Anna-Lena Kalteis, Thomas Vollbrecht, Lisa Gloning, Renate Stirner, Nadja Henrich, Johannes R. Bogner, Rika Draenert

**Affiliations:** Department of Infectious Diseases, Medizinische Klinik und Poliklinik IV der Ludwig-Maximilians-University, Munich, Germany; Massachusetts General Hospital, United States of America

## Abstract

HIV evades CD8 T cell mediated pressure by viral escape mutations in targeted CD8 T cell epitopes. A viral escape mutation can lead to a decline of the respective CD8 T cell response. Our question was what happened after the decline of a CD8 T cell response and - in the case of viral escape – if a new CD8 T cell response towards the mutated antigen could be generated in a population not selected for certain HLA alleles. We studied 19 antiretroviral-naïve HIV-1 infected individuals with different disease courses longitudinally. A median number of 12 (range 2-24) CD8 T cell responses towards Gag and Nef were detected per study subject. A total of 30 declining CD8 T cell responses were studied in detail and viral sequence analyses showed amino acid changes in 25 (83%) of these. Peptide titration assays and definition of optimal CD8 T cell epitopes revealed 12 viral escape mutations with one de-novo response (8%). The de-novo response, however, showed less effector functions than the original CD8 T cell response. In addition we identified 4 shifts in immunodominance. For one further shift in immunodominance, the mutations occurred outside the optimal epitope and might represent processing changes. Interestingly, four adaptations to the virus (the de-novo response and 3 shifts in immunodominance) occurred in the group of chronically infected progressors. None of the subjects with adaptation to the changing virus carried the HLA alleles B57, B*58:01 or B27. Our results show that CD8 T cell responses adapt to the mutations of HIV. However it was limited to only 20% (5 out of 25) of the epitopes with viral sequence changes in a cohort not expressing protective HLA alleles.

## Introduction

Developing a human immunodeficiency virus (HIV) vaccine is critical to end the pandemic with currently 33 million HIV infected people worldwide. A protective vaccine is still out of reach due to the virus’ effective strategies to evade immune pressure. A therapeutic vaccine that leads to better control of viremia in infected subjects would be an alternative. In some cases, therapeutic vaccines aim at inducing very specific immune responses e.g. towards certain CD8 T cell epitopes [1,2].

Vaccinating HIV-1 infected individuals could cause immune interferences that need to be considered beforehand. Their immune system has already been confronted with a certain viral sequence and mounted CD8 T cell responses specifically towards the infecting virus. Due to the diversity of HIV clones and adaptation to their hosts, any given vaccine sequence will differ from the autologous virus. One critical question in this setting is whether the immune system can create a new response towards the variant epitope. An impaired response to a second antigen has been termed “original antigenic sin” (OAS), first used to describe antibody responses to influenza virus. After an initial infection, re-infection with a second, new influenza strain boosted antibodies specific for the first infecting strain but not the second [3,4]. This concept was extended to CD8 T cell responses in 1998 [5] and to other viral infections like HCV [6,7]. A recent publication however failed to support OAS in a *Listeria monocytogenes* mouse model and the H-2K^b^-restricted CD8 SIINFEKL epitope and its variants [8]. 

A parallel to the two different viral sequences in the setting of vaccinating an already infected individual would be the development of viral escape mutations in the course of HIV infection. Studying this situation, it has been shown that HLA-B*57+ children can mount a new CD8 T cell response after the occurrence of a viral escape mutation within the Gag epitope TSTLQEQIGW [9]. In addition, there are reports on a single de-novo response after the development of viral escape in adults for a HLA-A*11 restricted epitope [10] and towards HLA-B*57 restricted epitopes in Gag [11-14] as well as HLA-B*27 restricted epitopes in Gag and Env [15-17]. More recently it has been shown in a single individual that de-novo responses can be mounted against low frequency escape mutations when studying the very early phase of infection [18]. It has been hypothesized that the ability to mount strong CD8 T cell responses to the mutating virus is one of the mechanisms for the often long-term control of viremia in HLA-B*57/B*58:01+ and HLA-B*27+, chronically HIV infected individuals. However it is unclear how frequently this occurs in the course of HIV infection when looking at a larger population not selected for certain HLA alleles and not concentrating on the extreme early phase of acute infection. With the present study we addressed this question by studying HIV-1 infected, treatment-naïve individuals longitudinally. We identified viral escape mutations within CD8 T cell epitopes towards Gag, Pol and Nef and tested for de-novo responses towards the new viral sequences. Our results show that CD8 T cell responses can adapt to ongoing evolution of HIV in a cohort not expressing protective HLA alleles.

## Methods

Definition of major keywords

Wild-type peptide: amino acid sequence of autologous virus at the first time point

Mutated peptide: amino acid sequence of autologous virus at the second time point with amino acid changes including mixed bases

The definition of viral escape mutation, shift in immunodominance and de-novo response was drawn by comparing Elispot peptide titration assays of wildtype and mutated peptide of first and second time point.

Viral escape mutation: at the first time point the mutated peptide was less (>2 log) recognized than the wildtype peptide.

Shift in immunodominance: at the second time point recognition of the mutated peptide exceeded recognition of the wildtype peptide in magnitude. This phenomenon was rather due to cross-recognition than to recruitment of a new CD8 T cell clone.

De-novo response: a new CD8 T cell response towards the mutated peptide arised at the second time point. In this case we hypothesize that a new CD8 T cell clone was recruited. 

### Study subjects

19 untreated HIV-infected individuals were chosen from a cohort that was studied longitudinally. Specific emphasis was laid on the fact that the chosen study subjects had declining CD8 T cell responses over the disease course. Study subjects were classified as following: A = early infected (n=7) (first presentation <12 months after infection with HIV, irrespective of CD4 count or viral load), C = controllers (n=4) (chronically infected, CD4 count >400/µl and VL <10.000 copies/ml) and P = progressors (n=8) (chronically infected, CD4 count <400/µl and VL >10.000 copies/ml) – both in the absence of antiretroviral treatment. All study subjects were antiretroviral naïve for the duration of the project. Subjects P05 and P09 were diagnosed as HIV positive within 12 months prior to first blood draw; clinical evaluation however suggested a chronic disease stage. Pol data from subjects P07, P08 and P09 was derived from a different project and only included if viral escape was proven; testing for Gag and Nef was not performed in these subjects [19]. For clinical data of study subjects see Table 1. The study was approved by the Institutional Review Board of the Ludwig-Maximilians-Universität, Munich. All study subjects participated after signing informed consent. High resolution HLA typing from extracted DNA was performed by the department for transfusion medicine of the University of Munich (HLA types of subjects epitope mapping was done for: A09: HLA-A*02:01, HLA-A*03:01, HLA-B*35:03, HLA-B*40:01, HLA-C*03:04, HLA-C*04:01; C02: HLA-A*02:01, HLA-A*32:01, HLA-B*08:01, HLA-B*18:01, HLA-C*07:01; P02: HLA-A*03:01, HLA-A*25:01, HLA-B*35:3, HLA-B*39:01, HLA-C*04:01, HLA-C*12:03; P05: HLA-A*24:02, HLA-A*31:01, HLA-B*35:08, HLA-B*35:01, HLA-C*04:01; P08: HLA-A*01:01, HLA-B*37:01, HLA-B*44:02, HLA-C*05:01, HLA-C*06:02).

**Table 1:** Clinical data of study subjects.

**Subject**		**CD4 count 1^st^ (/µl)**		**CD4 count 2^nd^ (/µl)**	**Viral load 1^st^ (cp/ml)**	**Viral load 2^nd^ (cp/ml)**	**Age (years)**		**Time since diagnosis (days)**		**Time between1^st^/ 2^nd^ (days**)
A01	752		779		52.085	62.990	37	76		196	
A02	508		263		871	19.493	48	17		504	
A03	692		485		30.369	15.146	22	112		530	
A05	753		756		16.677	43.169	42	317		462	
A06	448		162		106.337	167.579	25	173		571	
A07	1.327		417		335.211	40.829	27	5		1062	
A09	338		457		30.985	10.179	30	194		427	
C01	538		462		4.156	5.887	32	1.743		652	
C02	592		522		4.096	2.415	46	649		1577	
C03	785		761		3.739	8.321	37	3.553		236	
C04	517		543		2.247	5.589	56	2.273		1212	
P01	421		392		27 805	8.324	50	2.778		719	
P02	457		132		489.978	210.515	67	791		637	
P03	489		357		133.400	182.230	56	3.679		181	
P05	373		143		30.834	24.800	36	49		399	
P06	440		207		46.354	55.657	62	784		202	
P07*	464		509		135.364	103.725	46	502		692	
P08*	294		239		8.117	129.618	34	583		659	
P09*	275		318		33.517	74.744	59	194		238	

Asterisk (*): data derived from a different project as indicated in the methods section; cp/ml = copies/ml

Time since diagnosis = timing of first sampling

### Peptides

Synthetic overlapping peptides were used for screening (15-20 aa long, overlap of 5-10 aa; Gag: HIV-1 SF-2, Nef: HIV-1 Bru, NIBSC, England; Pol clade B consensus sequence of 2001 and Gag/Nef/Pol variants according to mutating viral sequences: EZBiolab, Carmel, USA). Truncations of longer peptides for epitope mapping were ordered as needed (EZBiolab, Carmel, USA) [20]. Peptides had a purity of ≥70%.

### Cell lines

EBV-transformed B lymphoblastoid cell lines (BLCL) were established in R20 medium (RPMI 1640; PAA, Pasching, Austria) supplemented with 2 mM L-glutamine, 50 U/ml penicillin, 50 mg/ml streptomycin, 10 mM Hepes and 20% heat-inactivated FCS (PAA)[21]. Antigen-specific CD8 T cell lines were generated from PBMC stimulated with synthetic peptide pulsed HLA matched BLCL in the presence of 20 million irradiated feeder cells in R10 medium (RPMI 1640 supplemented with 2 mM L-glutamine, 50 U/ml penicillin, 50 mg/ml streptomycin, 10 mM Hepes and 10% heat-inactivated FCS) supplemented with 100 IU/ml of recombinant IL-2 (ImmunoTools GmbH, Friesoythe, Germany).

### Interferon-gamma Elispot

HIV-specific CD8 T cell responses were quantified by Elispot assay using fresh or frozen PBMC (0.5 to 1x10^5^ per well) and peptides (final concentration: 12.5 µg/ml) as described previously [22]. Gamma interferon-producing cells were counted by direct visualization on an AID Elispot Reader (Autoimmun Diagnostika GmbH, Strassberg, Germany) and were expressed as spot-forming cells (SFC) per 10^6^ PBMC. Negative controls had to have ≤5 spots. Wells were counted as positive if there were ≥50 SFC/10^6^ PBMC. 2000 SFC per 10^6^ PBMC was chosen as upper cut-off.

Screening was done for CD8 T cell responses towards overlapping peptides spanning the HIV proteins Gag and Nef (Pol in subjects P07, P08, P09). CD8 T cell responses were defined as declining at a difference of >40% between first and second time point. 

If viral sequence analyses showed amino acid changes, peptide titration assays were performed comparing the recognition of wildtype and newly arising sequences. If the mutated peptide was equally or better recognized than the wildtype one at the first time point we considered it not as viral escape [23]. It could still represent a processing escape, however with our methodology we were not able to ascertain this. When, however, at the first time point the mutated peptide was less (>2 log) recognized than the wildtype peptide, we defined that finding as viral escape mutation. Due to the variability of Elispot assays, we chose >2 log difference as our cut-off. When with the appearance of the mutated antigen in the blood, recognition of the corresponding variant exceeded recognition of the original peptide in magnitude at the late time point, we defined this finding as a “shift in immunodominance”. For the identified de-novo responses and shifts in immunodominance, epitope mapping was performed as described [24]. Epitope predictions were done using the “SYFPEITHI” program and predicted optimal epitopes were compared experimentally to truncations with added or subtracted amino acids at the C-Terminal and the N-terminal position in Elispot assay using either PBMC or CD8 T cell lines. The optimal peptide was defined as the peptide that induced 50% maximal specific interferon-gamma production by T cells at the lowest peptide concentration [24]. For peptide titration assays, peptides were used at concentrations of 12.5 µg/ml - 1.25x10^-4^ µg/ml using 10-fold dilutions. Peptide comparisons were done in ≥2 independent experiments.

Intracellular cytokine staining for HLA restriction of CD8 T cell epitopes and for CD8 T cell effector functions

For determination of HLA class I restriction of CD8 T cell responses, intracellular cytokine staining assays for interferon-gamma using autologous or partly matched BLCL were performed as described [25]. For the Gag 21-29 (LRPGGKKRY) response in subject P08, the HLA class I restriction assay could not be performed due to poor CD8 T cell line growth. The cells were analysed using a FACS Calibur Flow Cytometer (BD Biosciences, Heidelberg, Germany) and FlowJo (Tree Star Inc., Ashland, USA) software. For negative controls, cells were incubated with BLCL without peptide, but were otherwise treated identically. Assays were done in ≥2 independent experiments.

For further evaluation of the quality of Pol 702-717 (wildtype and mutation)-specific CD8 T cell responses of subject P08, multicolour intracellular cytokine staining assays were performed as described [26]. Polyfunctionality of CD8 T cells was tested by incubating CD8 T cells of study subject P08 with the corresponding peptides and subsequent staining for CD8-pacific blue, gamma interferon-APC (BioLegend, San Diego, USA), TNF-alpha-PE/Cy7, IL2-PE and CD107a-FITC (BD Biosciences, Heidelberg, Germany). Cells were analysed using a FACSCanto Flow Cytometer (BD Biosciences, Heidelberg, Germany) and FlowJo (Tree Star Inc., Ashland, USA) software. A positive response was defined as at least twofold above background.

### Bulk sequencing of autologous virus with viral RNA

Viral RNA was extracted from the patients’ plasma with the QIAamp RNA Viral Mini Kit (Qiagen, Hilden, Germany) according to the manufacturer’s protocol. Viral RNA was transcribed into cDNA by using the SuperScript II First-Strand Synthesis System for reverse transcriptase PCR (Invitrogen, Darmstadt, Germany) and specific primers. DNA was amplified using nested PCR and specific primers. For some of the patients, individual primers were designed using OligoExplorer Software (Gene Link, Hawthorne, USA). For a detailed list of used primers please see Table S1. PCR cycling conditions were as follows: 97°C for 30 s, 32 cycles of 10 s at 97°C, 30 s at 56-60°C, 40 s at 72°C, and a final extension of 72°C for 10 min. PCR products were sequenced bidirectionally by Eurofins MWG, Martinsried, Germany. BioEdit (Ibis Therapeutics, Carlsbad, USA) was used to edit and align sequences. Completely changing peaks in sequencing histograms were counted as a change in sequence, whereas mixed peaks were counted as mixed bases and indicated with small instead of capital letters. Mixed bases were counted as arising amino acid changes as it signifies that at least part of the viral population developed a different sequence.

### Nucleotide sequence accession numbers

All sequences have been submitted to GenBank: KF692313 - KF692354.

## Results

### Declining CD8 T cell responses in HIV infected treatment-naïve individuals

**Figure 1:**
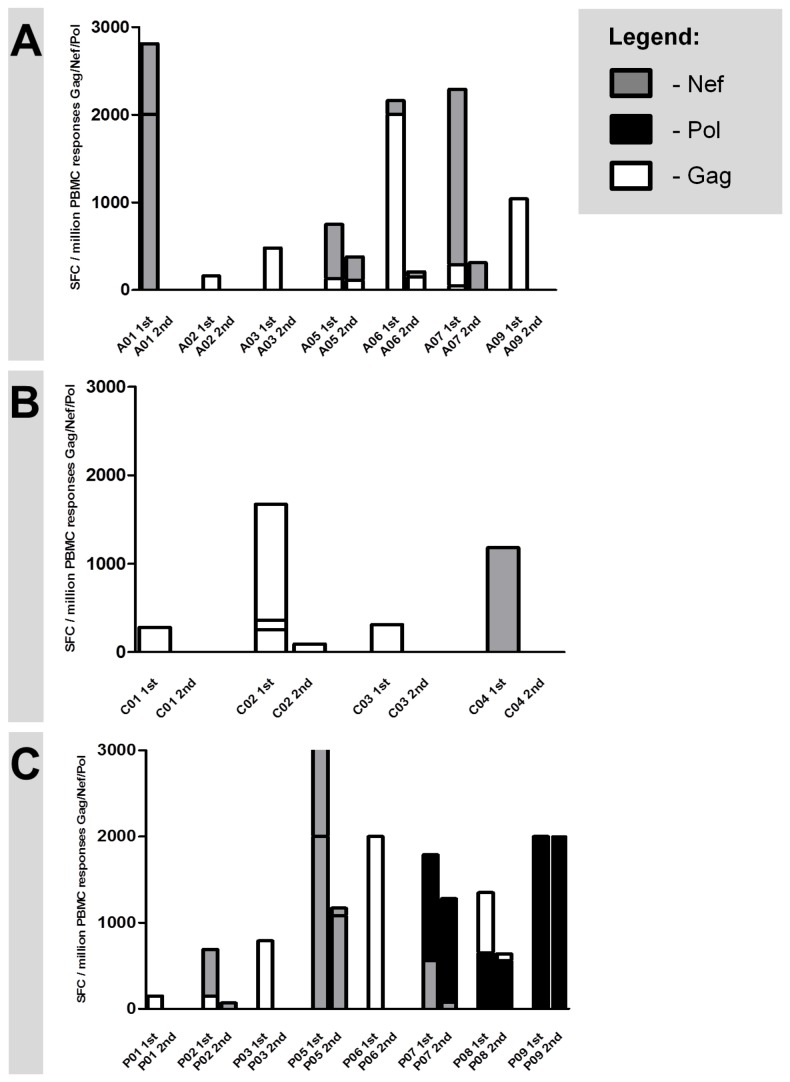
HIV-specific CD8 T cell responses. Overview of longitudinal changes in magnitude of HIV-specific CD8 T cell responses towards overlapping screening peptides spanning Gag (white bars), Pol (black bars) and Nef (grey bars) in 7 early infected individuals (A), 4 chronically infected controllers (B) and 8 chronically infected progressors (C) as measured by Elispot assay. Exact peptide denomination follows in Figure 2. Only responses are depicted where sequencing of autologous virus was done subsequentially. X-axis: first and second time point of each individual. Y-axis: magnitude of CD8 T cell responses expressed in spot forming cells per million PBMC.

HIV-infected treatment-naïve individuals were studied longitudinally (for >6 months; Table 1) and screened for CD8 T cell responses towards Gag and Nef by interferon-gamma Elispot. Next we aimed to identify those CD8 T cell responses that were subject to viral escape mutations. Due to the well-known correlation between declining CD8 T cell responses and viral escape mutations in the respective epitopes [27-32], we concentrated on CD8 T cell responses that declined over the disease course. The 19 study subjects had a median number of 12 CD8 T cell responses towards Gag and Nef (range 2-24). Median magnitude of chosen CD8 T cell responses was 650 SFC/10^6^ PBMC (range 50–2000 SFC/10^6^ PBMC) at the first time point and declined to a median magnitude of 0 SFC/10^6^ PBMC (range 0–2000 SFC/10^6^ PBMC) at the second time point (Figure 1). We chose 30 mostly declining CD8 T cell responses of the 19 subjects for further analysis. Exception for declining CD8 T cell responses is subject P09 (Figure 1C), where data was derived from a different project and viral escape was already proven [19].

### Longitudinal follow-up reveals viral sequence changes in the majority of studied CD8 T cell regions

**Figure 2:**
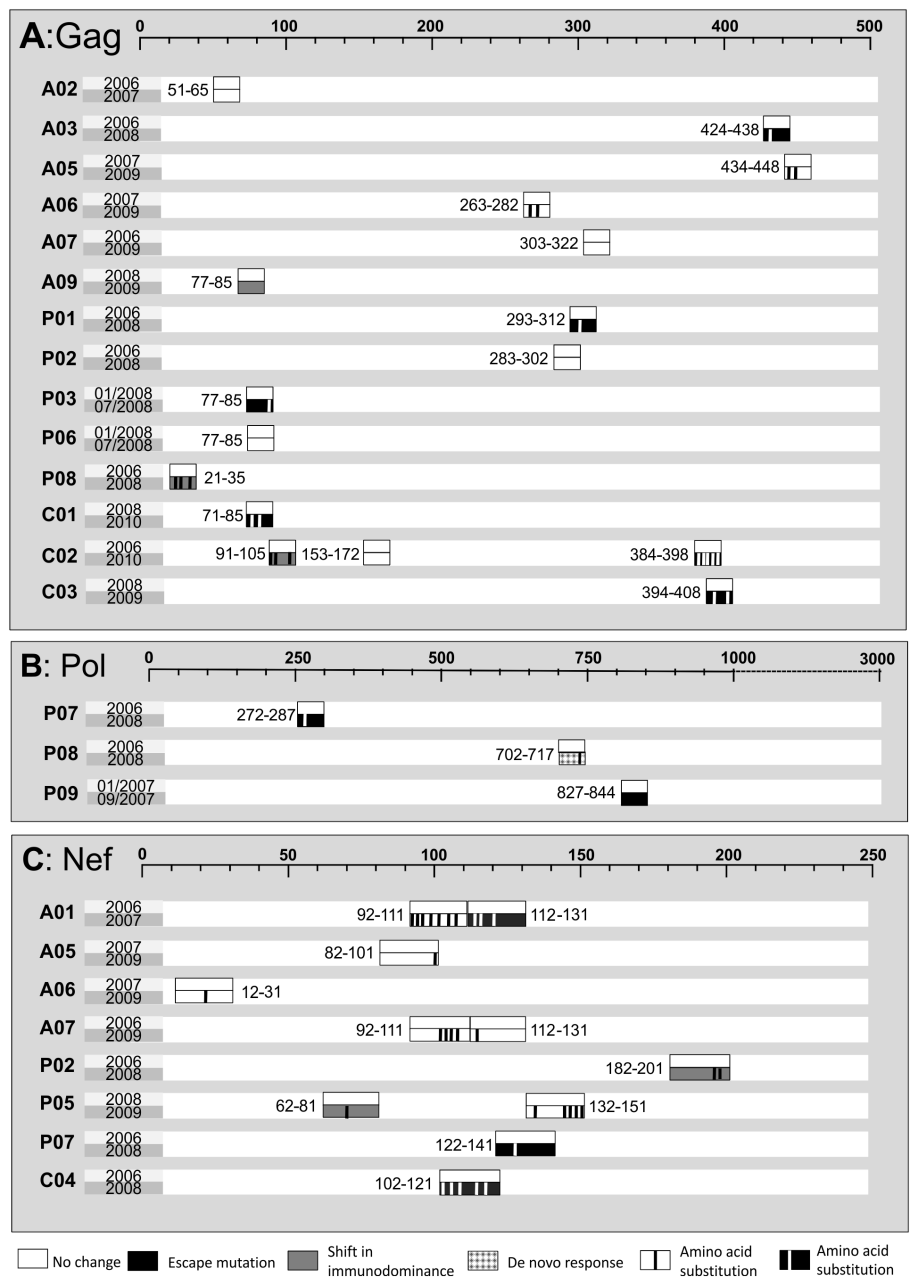
Sequencing results of autologous virus of regions in HIV-proteins Gag, Pol and Nef. Top scale indicates amino acid position according to HXB2 sequence of Gag (A), Pol (B) and Nef (C). On the left side are depicted: ID of individuals and years of consecutive blood draws. Boxes indicate the sequenced region and numbers next to boxes the exact amino acid position. 1 bar in a box stands for 1 amino acid substitution (including mixed bases) that either does not change the immune response (white box) or leads to viral escape (black box), a shift in immunodominance (grey box) or a de-novo response towards an escape mutation (checked box). For sequences in detail: supporting information Table S2.

The genetic variability of HIV can lead to viral escape from CD8 T cell recognition [28,29,33]. Sequencing of the autologous virus of the first and second time point was done for the chosen 30 regions (Figure 2 and Table S2). The CD8 T cell responses located in Nef showed amino acid changes in 11 out of 11 (100%) sequences over time. Viral sequence analyses of Gag showed amino acid changes in only 10 out of 16 (62.5%) CD8 T cell responses we had focused on. In individual A09 the amino acid changes in the well-known HLA-A*02:01 restricted Gag 77-85 (SLYNTVATL) were already present at the first time point. As indicated in the methods section, data for Pol was chosen by proven escape mutation. In total, 83% of the 30 regions experienced amino acid mutations over time.

### Viral escape mutations lead to one de-novo CD8 T cell response

**Figure 3:**
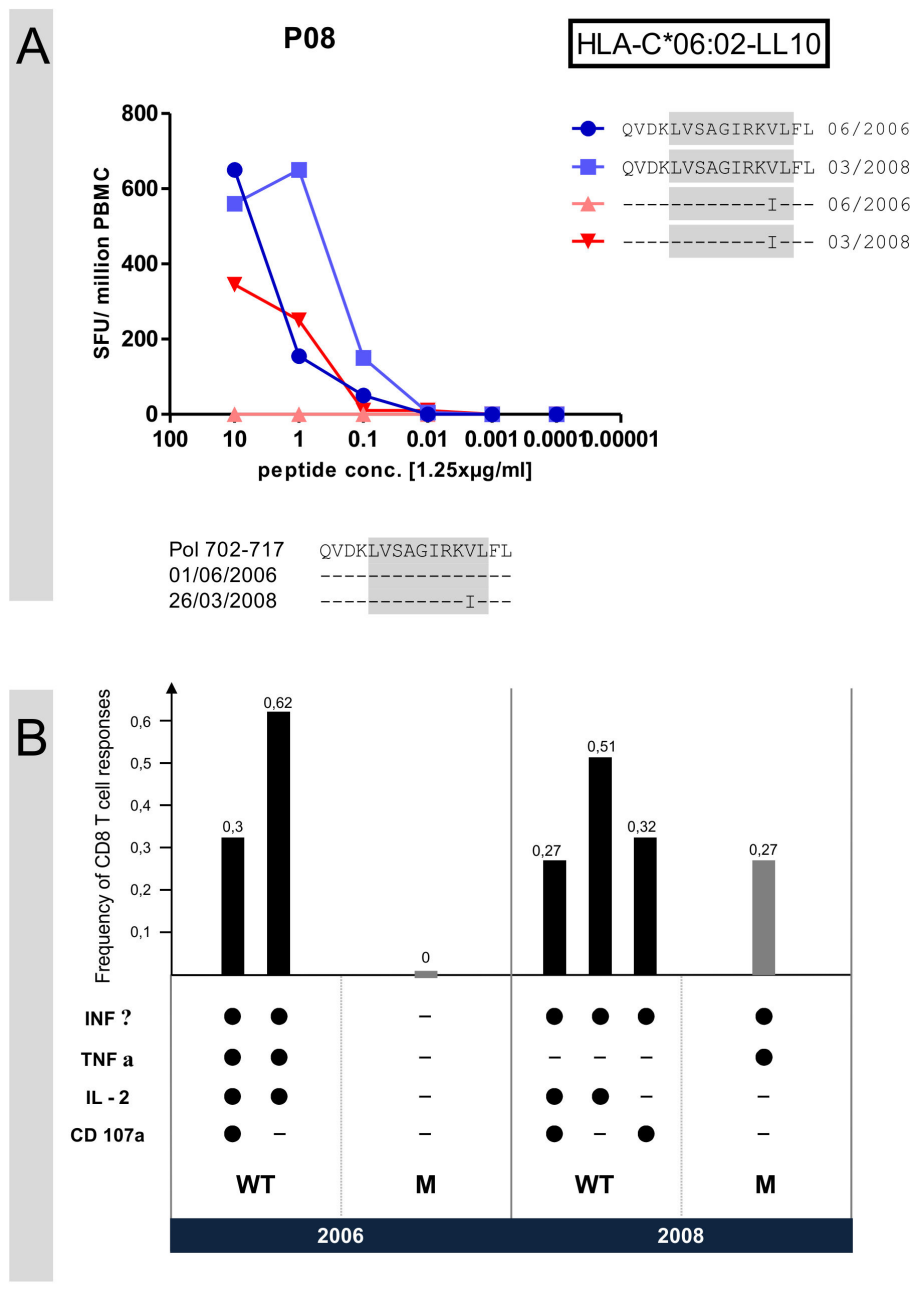
de-novo response of subject P08. (A) In blue wildtype peptide recognition, in red mutated peptide recognition at first and second time point as measured by titration Elispot assays. X-axis: peptide concentration in increasing dilution. Y-axis: magnitude of CD8 T cell response expressed in spot forming cells per million PBMC. Below the graphs are shown amino acid sequences of consensus sequence and autologous virus‘sequences of first and second time point. The optimal HLA-C*06:02 restricted epitope Pol 706-715 (LVSAGIRKVL) is highlighted in grey. Values are presented as mean values. (B) Evaluation of different effector functions towards the two different peptides. Bars represent the total CD8 T cell frequency to Pol 706-715 in subject P08 expressing the particular combination of functions shown. WT: recognition of wildtype peptide (Pol 706-715); M: recognition of mutated peptide (Pol 706-715; V714I). Left side: blood samples from early time point (2006), right side: blood samples from late time point (2008).

De-novo responses to a mutating CD8 T cell epitope are possible in HIV-infection [17,9-16]. For all arising amino acid mutations, we compared the recognition of wildtype and mutated sequence in peptide titration assays for the early and late time point using the longer overlapping screening peptides. By this method, we identified viral escape in 12 of 25 recognized peptides with amino acid mutations (48%). Furthermore we observed in subject P08 one de-novo response to the mutated region (Figure 3A). Epitope mapping for this CD8 response revealed the HLA-C*06:02 restricted Pol 706-715 (LVSAGIRKVL) as optimal epitope with the mutation at position 9 of the epitope (V714I). To our knowledge, this is the first time this epitope is described. In order to prove that we had indeed identified a de-novo CD8 T cell response, we studied samples of the early and late time point by looking for several effector functions. This analysis showed that there was production of interferon-gamma, TNF-alpha, IL-2 and CD107a after stimulation with wildtype peptide (0.3%) but no effector function after stimulation with mutated peptide for the early time point of P08. However we could show production of interferon-gamma, IL-2 and CD107a for wildtype peptide (0.27%) and production of interferon-gamma and TNF-alpha for stimulation with mutated peptide (0.27%) for the late time point (Figure 3B).

Taken together, we observed a de-novo response towards the mutated epitope in one out of 12 (8%) epitopes where a viral escape mutation had occurred.

### Viral sequence variations also lead to shift in immunodominance

**Figure 4:**
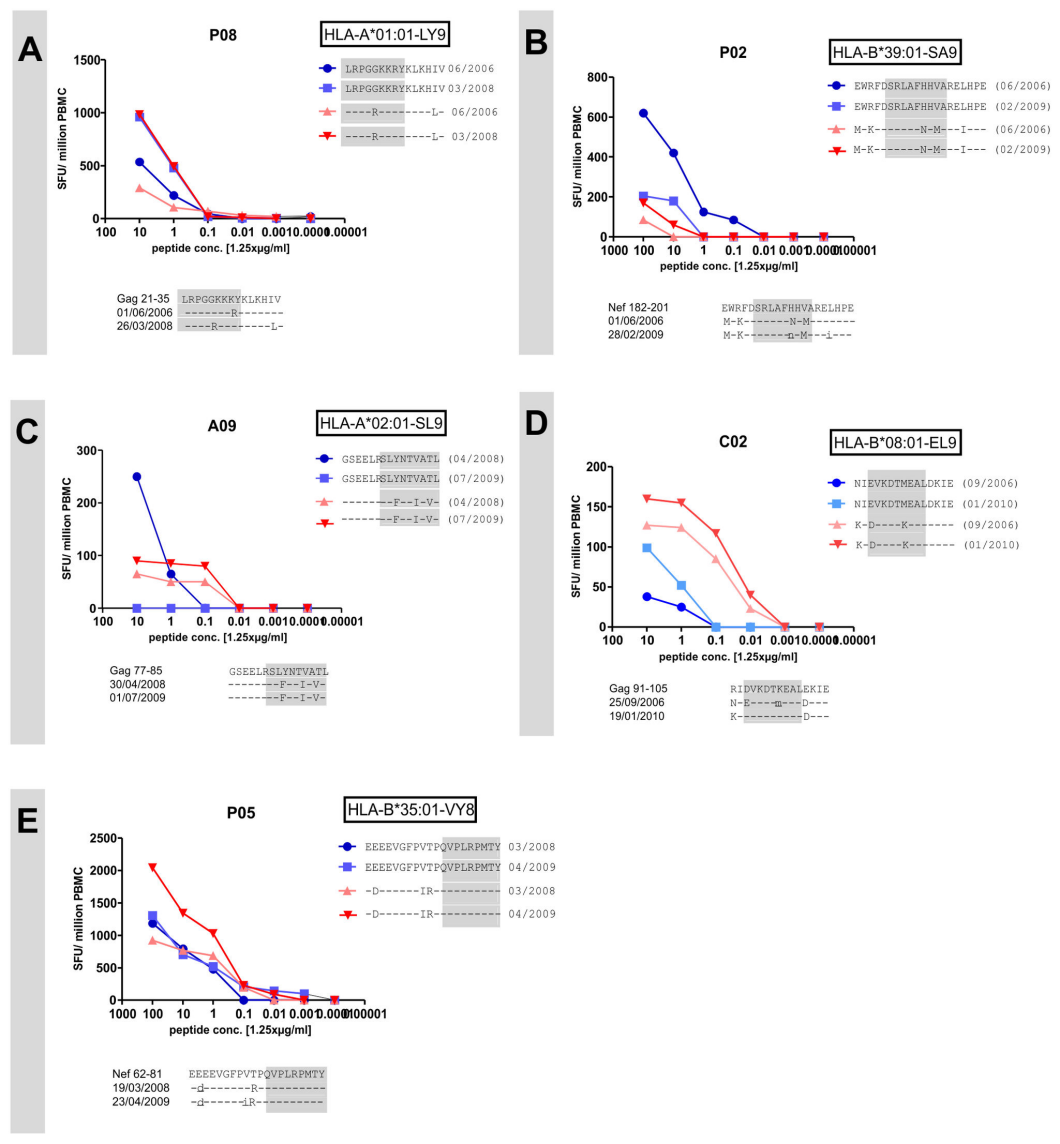
Shifts in immunodominance. In blue wildtype peptide recognition, in red mutated peptide recognition at first and second time point as measured by titration Elispot assays. X-axis: peptide concentration in increasing dilution. Y-axis: magnitude of CD8 T cell response expressed in spot forming cells per million PBMC. Below the graphs are shown amino acid sequences of consensus sequence and autologous virus‘sequences of first and second time point. The optimal epitopes are highlighted in grey (P08: HLA-A*01:01 restricted Gag 21-29 (LRPGGKKRY); P02: HLA-B*39:01 restricted Nef 187-195 (SRLAFNHMA); A09: HLA-A*02:01 restricted Gag 77-85 (SLYNTVATL); C02: HLA-B*08:01 restricted Gag 93-101 (EVKDTMEAL); P05: HLA-B*35:01 restricted Nef 74-81 (VPLRPMTY). Values are presented as mean values.

It has been demonstrated that HIV-specific CD8 T cells can cross-recognize mutant variants after the occurrence of a viral escape mutation [16,34]. In 5 of 25 (20%) mutating regions we observed a shift in magnitude of the CD8 T cell response between wildtype and mutated peptide. Epitope mapping was performed for these 5 regions subsequently. For P08 the optimal epitope was Gag 21-29 (LRPGGKKRY) (Figure 4A), this epitope has not been described before in literature. Epitope prediction programs predict this epitope to be restricted by HLA-A*01:01. For P02, epitope fine mapping revealed the HLA-B*39:01 restricted epitope Nef 187-195 (SRLAFNHMA) (Figure 4B). For A09 the optimal epitope is the well-known HLA-A*02:01 restricted Gag 77-85 (SLYNTVATL) (Figure 4C) [35]. As discussed above, here the amino acid mutations were already present in the autologous virus at the first time point. Therefore we hypothesized that the mutations occurred prior to the first blood draw. For C02, we identified the HLA-B*08:01 restricted Gag 93-101 (EVKDTMEAL) as the optimal epitope (Figure 4D). Interestingly, here the virus evolved towards the consensus sequence. 

In subject P05, epitope fine-mapping revealed the well-known HLA-B*35:01 restricted Nef 74-81 (VPLRPMTY) as the optimal epitope [36]. For this epitope, the detected escape mutation was located outside the epitope, preceding the optimal epitope (Figure 4E). Sequence analysis of this individual did not show amino acid changes within the epitope as described by others [37]. Viral mutations within the flanking region of the epitope can influence protein processing. In this case, however, we should not be able to detect differences using peptides loaded from externally onto the HLA-molecule as this usually occurs in Elispot assay. The result therefore remains unclear and we did not include it in our counting of adapting immune responses.

Overall, the CD8 T cell response adapted to the mutating antigen in 5 of 25 (20%) mutating regions (1 de-novo response and 4 shifts in immunodominance). 

## Discussion

In this study we addressed the question concerning the frequency of adaptation of the CD8 T cell response towards the rapidly mutating HIV in a cohort not selected for a certain HLA allele. We observed an adaptation towards viral escape mutations in 20% of the mutating regions targeted by CD8 T cell responses, either as a de-novo response or as a shift in immunodominance.

To study adapting CD8 T cell responses towards the mutating antigen we aimed to identify individuals with arising viral escape mutations. Due to the known correlation between viral escape mutations and the decline of respective CD8 T cell responses [10,27-31] we focused on declining CD8 T cell responses over the disease course. In contrast to other studies we did not focus on individuals expressing protective HLA alleles [9,11-16] or exclusively on acute HIV infection [10,18,29,30,38]. We purposefully included untreated, chronically infected individuals because viral escape occurs also in this stage of infection and this group has not been studied as extensively as acutely infected individuals [28,39]. 

De-novo responses to viral escape mutations exist and have been shown particularly well for HLA-B*57 restricted epitopes in Gag in adults and children [9,11-14]. Additionally there are reports about de-novo responses towards HLA-B*27 restricted epitopes [15,16]. It has been suggested that this is one of the mechanisms for the often long-term control of viremia in HLA-B*57/B*58:01+ individuals [9,11-14,17]. Recently adaption to changing viral sequences has been shown in subjects with acute HIV infection [18,38]. The mentioned studies find de-novo responses in higher frequency than our study. The plasticity of the immune system and therefore the ability to adapt to the changing virus seems to be greater in acute than in chronic infection. Interestingly, in our findings the shifting responses towards the mutant antigens where partly restricted by HLA-alleles associated with faster progression to AIDS (HLA-B*35:01, HLA-A*01) [40].

Not surprisingly we observed amino acid mutations in 100% of the Nef regions we had chosen, compared to only 62.5% in the Gag region. This is in concordance with the literature, where Gag is described as the more conserved region within the HIV polyprotein [41,42]. A limitation of bulk sequencing - compared to e.g. the deep sequencing methodology - is its relative insensitivity, where just the major base exchanges and therefore the predominant quasispecies of the virus can be observed. This could also serve as an explanation why some CD8 T cell responses decreased when no amino acid changes could be observed in bulk sequencing. However even with this method, we identified non-synonymous mutations in 83% of the chosen regions. The insensitivity of the bulk sequencing method does imply, however, that observed mutations are those of predominant quasispecies in the blood.

Viral escape from CD8 T cell mediated immune pressure occurs through the selection of sequence variations within epitopes crucial for HLA-binding or TCR recognition [27,28,39,43]. This form of viral escape has the result that the recognition of the variant epitope by CD8 T cells is reduced or lost. On the other hand viral escape mutations can influence antigen processing - a form of escape that is not fully detected by Elispot assays where peptides are loaded on the cells from externally [44-46]. This variant of viral escape is likely underestimated in our study. For 8 out of 25 CD8 T cell responses we found amino acid mutations without evidence for escape using our methodology. As stated above viral mutations within the flanking region of the epitope can influence protein processing and consecutively epitope presentation [44-46]. In the case of the HLA-B*35:01 restricted Nef 74-81 (VPLRPMTY), however, we should not be able to detect differences using the Elispot assay, as peptides are loaded from externally onto the HLA-molecule. 

Surprisingly, it was the group of chronically infected progressors where the immune system showed the greatest plasticity to the evolving virus. The observed de-novo response as well as 3 out of 5 shifts in immunodominance occurred in individuals who by definition are the least able to control the infection. Looking at the results of the polyfunctionality assay might explain this alleged contradiction: the primary response is a polyfunctional response with 4 different effector functions upon stimulation. The de-novo response – occurring later in the disease course towards a variant virus – is a response with only two effector functions. This leads to the question of how effective a new CD8 T cell response can be in a chronically infected untreated individual as CD8 T cell responses with limited effector functions have been associated with poor viral control [26]. In this respect, individuals positive for the HLA class I allele B*57 might be special. CD8 T cell responses towards viral escape mutations have been described best in these subjects and it has been hypothesized that the capacity to mount T cell responses to the arising variant epitopes contributes to the control of HIV in these subjects [11,12,14]. Our study supports this hypothesis by demonstrating that in a cohort not expressing protective HLA alleles the adaptation occurs less often and with a dysfunctional de-novo CD8 T cell response. However, a link between the decline of CD8 T cell responses and disease progression cannot be drawn as this study is purely descriptive.

## Conclusion

Vaccinating already infected subjects can cause immune interferences that need to be considered beforehand. The present study shows that it is possible to induce new CD8 T cell responses even in the setting of an existing immune response towards the same epitope with a different sequence. De-novo responses, however, are rare whereas shifts in immunodominance occur more often. 

## Supporting Information

Table S1 **List of primers used for PCR.** As described in the methods section, viral RNA was extracted from the patients plasma and reverse transcribed (“reverse”). Nested PCR was performed using consecutively two sets of primers (indicated as “1^st^ run” and “2^nd^ run”). Asterisk: primer specifically designed for study subject.
(DOCX)

Table S2 **Sequencing results of autologous virus in detail.** Given are sequencing results of first and second time point of autologous virus. Mixed bases are indicated in small, underlined letters.
(DOCX)
