# Supplementary material for: Adaptation of CD8 T Cell Responses to Changing HIV-1 Sequences in a Cohort of HIV-1 Infected Individuals Not Selected for a Certain HLA Allele
Source: PLoS One. 2013 Dec 3;8(12):e80045. doi: 10.1371/journal.pone.0080045 (PMC3849264; doi:10.1371/journal.pone.0080045)
Supplement: Table S1 — List of primers used for PCR. As described in the methods section, viral RNA was extracted from the patients plasma and reverse transcribed (“reverse”). Nested PCR was performed using consecutively two sets of primers (indicated as “1st run” and “2nd run”). Asterisk: primer specifically designed for study subject. (DOCX) [file pone.0080045.s001.docx]

**Supporting information table S1: List of primers used for PCR**

| **Subject** | **Sequenced**  **region** | **Primer** | **Primer forward** | **Primer backward** |
| --- | --- | --- | --- | --- |
| **A01** | 92-111  Nef | **Reverse** | CTTGAAGCACTCAAGGCAAGCTTTATTG |  |
|  |  | **1^st^ run** | GTGGCAAGTGGTCAAAACG | TGATGAAATGCTAGGCGGC |
|  |  | **2^nd^ run** | GGAGCAATCACAAGTAGCA | CTTCCACACTAACACTTCTC |
| **A01** | 112-131  Nef | **Reverse** | TACCAGAGTCACACAACAGA |  |
|  |  | **1^st^ run** | GTGGCAAGTGGTCAAAACG | TGATGAAATGCTAGGCGGC |
|  |  | **2^nd^ run** | GGAGCAATCACAAGTAGCA | CTTCCACACTAACACTTCTC |
| **A02** | 51-65  Gag | **Reverse** | GTATGGATTTTCAGGCCC |  |
|  |  | **1^st^ run** | CTTAAGCCTCAATAAAGCTTGCCTTGA | TCTGGGTTCGCATTTTGGAC |
|  |  | **2^nd^ run** | GCGGAGGCTAGAAGGAG | GTGGGGTGGCTCCTTCT |
| **A03** | 424-438  Gag | **Reverse** | GCTGGTGATCCTTTCCATCC |  |
|  |  | **1^st^ run** | AGAGAAGGCTTTCAGCCC | GTATGGATTTTCAGGCCC |
|  |  | **2^nd^ run** | CCAAGGGGAAGTGACATAGC | TCCTTTAGTTGCCCCCCTATC |
| **A05** | 434-448  Gag | **Reverse** | GTATGGATTTTCAGGCCC |  |
|  |  | **1^st^ run** | AGAGAAGGCTTTCAGCCC | GTATGGATTTTCAGGCCC |
|  |  | **2^nd^ run** | CCAAGGGGAAGTGACATAGC | TACTGTATCATCTGCTCCTGTATC |
| **A05** | 82-101  Nef | **Reverse** | TACCAGAGTCACACAACAGA |  |
|  |  | **1^st^ run** | CATAATGATAGTAGGAGGCTTGG | TGAAGCACTCAAGGCAAGC |
|  |  | **2^nd^ run** | GTAAGGGAAAGAATGAGACG | TGATGAAATGCTAGGCGGC |
| **A06** | 263-282  Gag | **Reverse** | GTATGGATTTTCAGGCCC |  |
|  |  | **1^st^ run** | GCGGAGGCTAGAAGGAG | GGGAAGGCCAGATCTTCC |
|  |  | **2^nd^ run** | TCAGCCCAGAAGTGATACCC | GGGTTCGCATTTTGGACC |
| **A06** | 12-31  Nef | **Reverse** | TACCAGAGTCACACAACAGA |  |
|  |  | **1^st^ run** | TAGGAGGCTTGGTAGGTTTA | GTTGTTCTCTCCTTCATTGG |
|  |  | **2^nd^ run** | ATTGTGGAACTTCTGGGACG | TCTTTGGGAGTGAATTAGCC |
| **A07** | 303-322  Gag | **Reverse** | GCTGGTGATCCTTTCCATCC |  |
|  |  | **1^st^ run** | CCAAGGGGAAGTGACATAGC | GTATGGATTTTCAGGCCC |
|  |  | **2^nd^ run** | TCCACCTATCCCAGTAGG | GTATGGATTTTCAGGCCC |
| **A07** | 92-111  Nef | **Reverse** | TACCAGAGTCACACAACAGA |  |
|  |  | **1^st^ run** | CATAATGATAGTAGGAGGCTTGG | TGAAGCACTCAAGGCAAGC |
|  |  | **2^nd^ run** | GTAAGGGAAAGAATGAGACG | GTTGTTCTCTCCTTCATTGG |
| **A07** | 112-131  Nef | **Reverse** | TACCAGAGTCACACAACAGA |  |
|  |  | **1^st^ run** | CATAATGATAGTAGGAGGCTTGG | TGAAGCACTCAAGGCAAGC |
|  |  | **2^nd^ run** | GTAAGGGAAAGAATGAGACG | GTTGTTCTCTCCTTCATTGG |
| **A09** | 77-85  Gag | **Reverse** | GTATGGATTTTCAGGCCC |  |
|  |  | **1^st^ run** | CTTAAGCCTCAATAAAGCTTGCCTTGA | GGGTTCGCATTTTGGACC |
|  |  | **2^nd^ run** | GCGGAGGCTAGAAGGAG | GTGGGGTGGCTCCTTCT |
| **C01** | 71-85  Gag | **Reverse** | GTATGGATTTTCAGGCCC |  |
|  |  | **1^st^ run** | CTTAAGCCTCAATAAAGCTTGCCTTGA | GGGTTCGCATTTTGGACC |
|  |  | **2^nd^ run** | GCGGCTGGTGAGTACGCC | GTGGGGTGGCTCCTTCT |
| **C02** | 91-105  Gag | **Reverse** | GTATGGATTTTCAGGCCC |  |
|  |  | **1^st^ run** | GCGACTGGTGAGTACGCC | GGGAAGGCCAGATCTTCC |
|  |  | **2^nd^ run** | ATATAGTATGGGCAAGCAGG | GTTCCTGCTATGTCACTTCC |
| **C02** | 153-172  Gag | **Reverse** | GTATGGATTTTCAGGCCC |  |
|  |  | **1^st^ run** | GCGACTGGTGAGTACGCC | GGGAAGGCCAGATCTTCC |
|  |  | **2^nd^ run** | ATATAGTATGGGCAAGCAGG | GTTCCTGCTATGTCACTTCC |
| **C02** | 384-398  Gag | **Reverse** | GTATGGATTTTCAGGCCC |  |
|  |  | **1^st^ run** | GCGACTGGTGAGTACGCC | GGGAAGGCCAGATCTTCC |
|  |  | **2^nd^ run** | TAGAAGACATGATGACAGCATG | CCTTCCTTTCCACATTTCCA |
| **C03** | 394-408  Gag | **Reverse** | GCTGGTGATCCTTTCCATCC |  |
|  |  | **1^st^ run** | GCGGAGGCTAGAAGGAG | TTCTGGTGGGGCTGTTGG |
|  |  | **2^nd^ run** | GATGACAGCATGTCAGGG | TTCTGGTGGGGCTGTTGG |
| **C04** | 102-121  Nef | **Reverse** | TACCAGAGTCACACAACAGA |  |
|  |  | **1^st^ run** | GTGGCAAGTGGTCAAAACG | TGATGAAATGCTAGGCGGC |
|  |  | **2^nd^ run** | GGAGCAATCACAAGTAGCA | CTTCCACACTAACACTTCTC |
| **P01** | 293-312  Gag | **Reverse** | GCTGGTGATCCTTTCCATCC |  |
|  |  | **1^st^ run** | GCGGAGGCTAGAAGGAG | GGGAAGGCCAGATCTTCC |
|  |  | **2^nd^ run** | AGAGAAGGCTTTCAGCCC | GGGTTCGCATTTTGGACC |
| **P02** | 283-302  Gag | **Reverse** | GCTGGTGATCCTTTCCATCC |  |
|  |  | **1^st^ run** | GCGGAGGCTAGAAGGAG | GGGAAGGCCAGATCTTCC |
|  |  | **2^nd^ run** | AGAGAAGGCTTTCAGCCC | GGGTTCGCATTTTGGACC |
| **P02** | 182-201  Nef | **Reverse** | TACCAGAGTCACACAACAGA |  |
|  |  | **1^st^ run** | TAAAAGAAAAGGGGGGACTG | CAACAGACGGGCACACACTA |
|  |  | **2^nd^ run** | TAAAAGAAAAGGGGGGACTG | TGAAGCACTCAAGGCAAGC |
| **P03** | 77-85  Gag | **Reverse** | GTATGGATTTTCAGGCCC |  |
|  |  | **1^st^ run** | CTTAAGCCTCAATAAAGCTTGCCTTGA | GGGTTCGCATTTTGGACC |
|  |  | **2^nd^ run** | AAATTTTGACTAGCGGAGGC | GTGGGGTGGCTCCTTCT |
| **P05** | 62-81  Nef | **Reverse** | TACCAGAGTCACACAACAGA |  |
|  |  | **1^st^ run** | GGGGCAGTATCTCGAGA | GCTCATAGGGTGTAATAAGC |
|  |  | **2^nd^ run** | *ACATGGAGCACTCACAAGT | GTTGTTCTCTCCTTCATTGG |
| **P05** | 132-151  Nef | **Reverse** | TACCAGAGTCACACAACAGA |  |
|  |  | **1^st^ run** | GGGGCAGTATCTCGAGA | GCTCATAGGGTGTAATAAGC |
|  |  | **2^nd^ run** | ACATGGAGCACTCACAAGT | GTTGTTCTCTCCTTCATTGG |
| **P06** | 77-85  Gag | **Reverse** | GTATGGATTTTCAGGCCC |  |
|  |  | **1^st^ run** | CTTAAGCCTCAATAAAGCTTGCCTTGA | GGGTTCGCATTTTGGACC |
|  |  | **2^nd^ run** | GCGGCTGGTGAGTACGCC | GTGGGGTGGCTCCTTCT |
| **P07** | 272-287  Pol | **Reverse** | CCCTTTTCTTTTAAAATTGTG |  |
|  |  | **1^st^ run** | AATTGGGCCTGAAAATCC | TCACTAGCCATTGCTCTCC |
|  |  | **2^nd^ run** | CTAAATTAGGAAAAGCAGG | TCACTAGCCATTGCTCTCC |
| **P07** | 122-141  Nef | **Reverse** | TTGATGCCGTTCTTCTGCT |  |
|  |  | **1^st^ run** | ACAGGCTGTTGTTCTCTCC | GATGTTGCCGTCCTCCTTG |
|  |  | **2^nd^ run** | GCCTCTTCTACCTTATCTGG | TTGATGCCGTTCTTCTGCT |
| **P08** | 21-35  Gag | **Reverse** | GTATGGATTTTCAGGCCC |  |
|  |  | **1^st^ run** | GCGGCTGGTGAGTACGCC | GGGTTCGCATTTTGGACC |
|  |  | **2^nd^ run** | GCGGCTGGTGAGTACGCC | ACTTCTGGGCTGAAAGCC |
| **P08** | 702-717  Pol | **Reverse** | CCCTTTTCTTTTAAAATTGTG |  |
|  |  | **1^st^ run** | GGGGCAAGGCCAATGGAC | CCCTTTTCTTTTAAAATTGTG |
|  |  | **2^nd^ run** | GTCAATACCCCTCCCTTAG | CCCTTTTCTTTTAAAATTGTG |
| **P09** | 827-844  Pol | **Reverse** | CCCTTTTCTTTTAAAATTGTG |  |
|  |  | **1^st^ run** | AATTGGGCCTGAAAATCC | TCACTAGCCATTGCTCTCC |
|  |  | **2^nd^ run** | CTAAATTAGGAAAAGCAGG | TCACTAGCCATTGCTCTCC |
