# Supplementary material for: Adaptation of CD8 T Cell Responses to Changing HIV-1 Sequences in a Cohort of HIV-1 Infected Individuals Not Selected for a Certain HLA Allele
Source: PLoS One. 2013 Dec 3;8(12):e80045. doi: 10.1371/journal.pone.0080045 (PMC3849264; doi:10.1371/journal.pone.0080045)
Supplement: Table S2 — Sequencing results of autologous virus in detail. Given are sequencing results of first and second time point of autologous virus. Mixed bases are indicated in small, underlined letters. (DOCX) [file pone.0080045.s002.docx]

**Supporting information table S2: Sequencing results of autologous virus in detail**

| **Subject** | **Sequenced**  **region** | **timepoint** | **Sequence** |
| --- | --- | --- | --- |
| **A01** | 92-111  Nef | 2006  2007 | KEKGGLEGLIHSQKRQDILD  rkeGGLdGLIySQkRQeILD |
| **A01** | 112-131  Nef | 2006  2007 | LWVYHTQGYFPDWQNYTPGP  LWvhHTQGFFPDWQNYTPGP |
| **A02** | 51-65  Gag | 2006  2007 | LETAEGCQQIIEQLQ  LETAEGCQQIIEQLQ |
| **A03** | 424-438  Gag | 2006  2008 | KDCNERQANFLGKIW  KDChERQANFLGKIW |
| **A05** | 434-448  Gag | 2007  2009 | LGKIWPSYKGRPGNF  LGKvWPSHKGRPGNF |
| **A05** | 82-101  Nef | 2007  2009 | KAAVDLSHFLKEKGGLEGLV  KAAVDLSHFLKEKGGLEGLI |
| **A06** | 263-282  Gag | 2007  2009 | KRWIIMGLNKVVRMYSPTSI  KKWIIMGLNKIVRMYSPTSI |
| **A06** | 12-31  Nef | 2007  2009 | DWPKVRERMRkIDPTAAADGVG  DWPKVRERMRRIDPTAAADGVG |
| **A07** | 303-322  Gag | 2006  2009 | TLRAEQASQDVKNWMTETLL  TLRAEQASQDVKNWMTETLL |
| **A07** | 92-111  Nef | 2006  2009 | KEKGGLEGLIYSQRRQDILD  KEKGGLEGLIySkkRQeILD |
| **A07** | 112-131  Nef | 2006  2009 | LWIYHTQGFFPDWQNYTPGP  LWvYHTQGFFPDWQNYTPGP |
| **A09** | 77-85  Gag | 2008  2009 | SLFNTIAVL  SLFNTIAVL |
| **C01** | 71-85  Gag | 2008  2010 | GSEELKSMxNTVAiL x=s/c/y  GSEELKSMFNTVAVL |
| **C02** | 91-105  Gag | 2006  2010 | NIEVKDTmEALDKIE  KIDVKDTKEALDKIE |
| **C02** | 153-172  Gag | 2006  2010 | NAWVKVVEEKAFSPEVIPMF  NAWVKVVEEKAFSPEVIPMF |
| **C02** | 384-398  Gag | 2006  2010 | KNQRKAIKCFNCGKE  RNQKRxVKCFNCGKE x=i/v |
| **C03** | 394-408  Gag | 2008  2009 | NCGQEGRIARNCRAP  NCGREGHIARNCRAP |
| **C04** | 102-121  Nef | 2006  2008 | YSKKRQEILDLWVHHTQGFF  WSKDRQDILDLWVYNTQGFF |
| **P01** | 293-312  Gag | 2006  2008 | FRDYVDRFYKTLRAEQASQE  FRDYVDQFYKTLRAEQASQE |
| **P02** | 283-302  Gag | 2006  2008 | LDIRQGPKEPFRDYVDRFYK  LDIRQGPKEPFRDYVDRFYK |
| **P02** | 182-201  Nef | 2006  2008 | MWKFDSRLAFNHMARELHPE  MWKFDSRLAFnHMAREiHPE |
| **P03** | 77-85  Gag | 01/2008  07/2008 | SLFNTIATL  SLFNTiATL |
| **P05** | 62-81  Nef | 2008  2009 | EdEEVGFPVRPQVPLRPMTY  EdEEVGFPiRPQVPLRPMTY |
| **P05** | 132-151  Nef | 2008  2009 | GiRHPLTFGWCFKLVPAEQD  GiRLPLTFGWCFKLVPVDRE |
| **P06** | 77-85  Gag | 01/2008  07/2008 | SLYNTVAVL  SLYNTVAVL |
| **P07** | 272-287  Pol | 2006  2008 | SVPLDKDFRKYTAFTI  SVPLDKNFRKYTAFTI |
| **P07** | 122-141  Nef | 2006  2008 | PDWQNYTPGPGVRFPLTFGW  PDWQNYTPGPGTRFPLTFGW |
| **P08** | 21-35  Gag | 2006  2008 | LRPGGKKRYKLKHIV  LRPGRKKKYKLKHLV |
| **P08** | 702-717  Pol | 2006  2008 | QVDKLVSAGIRKVLFL  QVDKLVSAGIRKILFL |
| **P09** | 827-844  Pol | 01/2007  09/2007 | VIHTDNGGNFTSGAVKAA  VIHTDNGGNFTSGAVKAA |
